# Development and Internal Validation of Machine Learning Algorithms to Predict 30-Day Readmission in Patients Undergoing a C-Section: A Nation-Wide Analysis

**DOI:** 10.3390/jpm15100476

**Published:** 2025-10-02

**Authors:** Audrey Andrews, Nadia Islam, George Bcharah, Hend Bcharah, Misha Pangasa

**Affiliations:** 1 Department of Obstetrics and Gynecology, Creighton University, Phoenix, AZ 85012, USA; audreycrawford@creighton.edu; 2 Mayo Clinic Alix School of Medicine, Phoenix, AZ 85054, USA; islam.nadia@mayo.edu (N.I.); sa205799@atsu.edu (H.B.); 3 Department of Obstetrics and Gynecology, ValleyWise Health, Phoenix, AZ 85008, USA; mishapangasa@creighton.edu

**Keywords:** Cesarean section, postpartum readmission, machine learning, predictive modeling, risk stratification, maternal health

## Abstract

**Background/Objectives**: Cesarean section (C-section) is a common surgical procedure associated with an increased risk of 30-day postpartum hospital readmissions. This study utilized machine learning (ML) to predict readmissions using a nationwide database. **Methods**: A retrospective analysis of the National Surgical Quality Improvement Project (2012–2022) included 54,593 patients who underwent C-sections. Random Forests (RF) and Extreme Gradient Boosting (XGBoost) models were developed and compared to logistic regression (LR) using demographic, preoperative, and perioperative data. **Results:** Of the cohort, 1306 (2.39%) patients were readmitted. Readmitted patients had higher rates of being of African American race (17.99% vs. 9.83%), diabetes (11.03% vs. 8.19%), and hypertension (11.49% vs. 4.68%) (*p* < 0.001). RF achieved the highest performance (AUC = 0.737, sensitivity = 72.03%, specificity: 61.33%), and a preoperative-only RF model achieved a sensitivity of 83.14%. Key predictors included age, BMI, operative time, white blood cell count, and hematocrit. **Conclusions**: ML effectively predicts C-section readmissions, supporting early identification and interventions to improve patient outcomes and reduce healthcare costs.

## 1. Introduction

Cesarean section (C-section) is currently the most commonly performed surgery worldwide and represents approximately one third of all deliveries in the United States [1]. Moreover, this rate does not appear to be declining despite efforts to reduce the rates of both primary and repeat C-sections [2]. The overall C-section rate in the United States increased from a rate of 20.7% in 1996 to a rate of 32.9% in 2009, representing a 60% increase in this time period. Between 2009 to 2019, the C-section rate slightly declined to 31.7% but again increased in 2020 (31.8%) and 2021 (32.1%) [3].

C-section itself is a significant risk factor for hospital readmission in the postpartum period, with one study finding that C-section increased the chance of readmission by 2.69 times compared to vaginal delivery [4]. In addition, women who have had a complicated C-section, as defined by reoperation, intraoperative organ injury, or surgical site complications, have been found to have a significantly higher overall readmission rate compared to uncomplicated surgeries [5].

There are many known risk factors and comorbidities associated with higher rates of postpartum readmission in general, including maternal medical conditions, multifetal pregnancy, prolonged labor, postpartum hemorrhage, fever, advanced maternal age, substance use disorders, social determinants of health as well as birth conditions such as C-section or operative birth [6]. That is why the creation of risk-stratification tools for postpartum complications, such as readmission, can aid in proactive management of high-risk patients in hopes to improve their C-section outcomes.

Machine learning (ML) and artificial intelligence serve as powerful tools that may improve the understanding of predictive metrics in all areas of medicine [7]. While numerous specialties have displayed the potential uses of ML in predictive modeling and risk-stratification, such literature in the field of obstetrics and gynecology is scarce. One study by Firouzbakht et al. implemented different machine learning models to predict postpartum readmission rates in Iranian women, however they did not report performance of models using AUC or confusion matrices nor compare the performance of the ML to other non-ML models [4]. Other studies in the field of obstetrics and gynecology have utilized machine learning and statistical models to predict postpartum hemorrhage and readmission for hypertensive disorders of pregnancy, however the research remains limited in the field [8,9]. This study aims to develop ML algorithms capable of predicting 30-day readmission in patients undergoing C-section using a large-scale, nationwide dataset. The study seeks to identify significant preoperative and perioperative predictors of readmission which could be utilized to inform clinical decision-making and optimize patient care.

## 2. Materials and Methods

### 2.1. Study Design and Data Source

This is a retrospective cohort analysis conducted using data from the American College of Surgeons National Surgical Quality Improvement Project (ACS NSQIP) database, Participant Use File (PUF) spanning the years 2012–2022. The NSQIP database is a nationally validated, risk-adjusted and outcomes-based program designed to measure the quality of surgical care [10]. It includes data from over 700 participating hospitals across the United States. The PUF is available to researchers at participating institutions under a standard data use agreement, and this study was conducted in compliance with the ACS NSQIP data use policies.

### 2.2. Study Population

The study population includes all women who underwent a C-section during the study period. Patients were identified using current procedural terminology (CPT) codes (59510, 59514, 59620, 59622). Inclusion criteria were women aged 18 years or older, those undergoing a primary or repeat C-section and availability of preoperative, perioperative and postoperative data in the NSQIP database. Exclusion criteria included missing key demographic or clinical variables.

### 2.3. Data Collection

Demographic, preoperative, and postoperative variables were extracted from the NSQIP database, including patient age, BMI, race, comorbid conditions, operative details, preoperative baseline conditions (Table 1) and postoperative characteristics (Table 2). The primary outcome of interest was a 30-day hospital readmission following a C-section.

The main outcome of interest was readmission, which was defined as unplanned readmission to the hospital within 30 days of discharge. Post-operative complications were defined as organ/surgical site infection, wound infection, dehiscence, pneumonia, reintubation, pulmonary embolism, failure to wean ventilation, urinary tract infection, bleeding requiring a transfusion (within 72 h), deep vein thrombosis, sepsis, return to operating room, and total hospital length of stay.

### 2.4. Machine Learning Model Development

Two machine learning models, Random Forest (RF) and Extreme Gradient Boosting (XGBoost), were developed and compared to logistic regression (LR) [11,12]. These models were chosen based on their ability to handle large complex datasets with numerous predictor variables. Both pre- and post-operative variables were initially used to create the models, and the best performing one was used to create a final model that is only based on pre-operative variables. The RF and XGBoost models that include postoperative variables offer a comprehensive retrospective analysis of risk factors leading up to readmission, while the latter pre-operative variables-only model is meant to support bedside prediction at or prior to discharge.

A multivariable LR model was developed as a baseline for comparison. The model was implemented using the glm function in R with a binomial family to predict the binary outcome of 30-day readmission. To ensure a direct and fair comparison, the LR model included the exact same set of predictor variables as the RF and XGBoost models.

Prior to model development, data preprocessing steps were applied to ensure model compatibility and reproducibility. Categorical variables were one-hot encoded using the dummyVars function from the caret package. Continuous variables, including laboratory values and vital signs, were centered and scaled to normalize the distributions across predictors. Records with missing data in key predictive fields were excluded from the analysis. These preprocessing steps were applied consistently across all machine learning models to ensure fairness in model comparisons.

The RF model was implemented using the “ranger” and “caret” packages in R. Hyperparameter optimization was performed using a grid search strategy with 10-fold cross-validation. The specific hyperparameters tuned included the number of variables randomly sampled at each split (mtry), with values tested at the square root of the number of predictors, 50% of the predictors, and the total number of predictors minus one. The node-splitting rule (splitrule) was varied between the “gini” and “extratrees” criteria. The minimum node size (min.node.size) was tested at values of 1, 5, and 10. The final model was selected based on the highest average area under the receiver operating characteristic (ROC) curve during cross-validation. Variable importance was assessed using the impurity-based method and visualized with bar plots. Classification performance on the held-out testing set was evaluated using confusion matrices to report sensitivity, specificity, precision, and F1 score, as well as ROC analysis to compute the AUC. Additionally, classification thresholds were optimized based on Youden’s index, and recall-precision trade-offs were assessed over a range of thresholds to evaluate model calibration and robustness.

The XGBoost model was constructed using the “xgboost” and “caret” packages. data was converted to a numeric matrix format as required by the xgboost training function. The following hyperparameters were tuned or specified: the learning rate (eta) was fixed at 0.1 to promote stable convergence; the maximum tree depth (max_depth) was tested in the range of 3 to 6, with 4 ultimately selected to reduce overfitting while maintaining complexity; and both row and column subsampling ratios (subsample and colsample_bytree) were set to 0.8 to introduce stochasticity. To address class imbalance, the scale_pos_weight parameter was set to the ratio of negative to positive class instances in the training data. The number of boosting rounds (nrounds) was set to 100 with an early stopping criterion of 10 rounds, using AUC as the evaluation metric. Model performance was assessed using ROC curves and AUC, and classification metrics such as precision, recall, and F1 score were derived from confusion matrices. Threshold optimization was again performed using Youden’s index to refine classification accuracy.

Feature importance for RF was calculated using the impurity-based criterion, specifically the mean decrease in Gini index, which measures the total reduction in node impurity attributed to each variable across all trees. Feature importance in XGBoost was computed using the default “gain” metric, which reflects the average improvement in the splitting criterion (log loss or classification error) brought by each feature across all trees.

The dataset was randomly split into training (80%) and testing (20%) sets. This split was stratified by the readmission outcome to ensure that the class distribution (2.39% readmitted) was preserved in both subsets. The training set was used for all model development, including hyperparameter tuning via 10-fold cross-validation, while the testing set was reserved for a single, final evaluation of the optimized models. Model performance was evaluated using confusion matrices (sensitivity, specificity) as well as the area under the curve (AUC) of the receiver operating characteristic (ROC) curve. The optimal classification threshold for converting model probabilities into binary predictions was determined using Youden’s J statistic (J = Sensitivity + Specificity − 1). This method identifies the threshold on the ROC curve that maximizes the vertical distance from the line of no-discrimination, thereby finding an optimal balance between the true positive rate and the false positive rate. DeLong’s method was used to impute confidence intervals (CIs) for the training and testing AUCs. Different models were compared using McNemar’s test, and continuity-corrected χ^2^ statistic and corresponding two-sided *p*-values were calculated. A *p*-value ≤ 0.05 indicates significance. R studio 2022.12.0 was used for statistical analysis.

## 3. Results

### 3.1. Final Cohort

**Figure 1:**
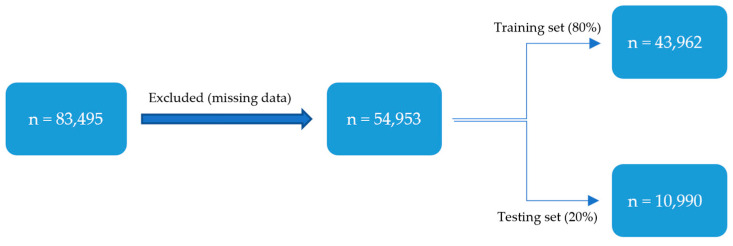
Cohort Selection and Splitting.

A total of 54,593 patients who underwent C-section between 2012 and 2022 were included in the study, of which 80% and 20% were split into the training and testing sets respectively (Figure 1). Of these, 1306 (2.39%) experienced a 30-day hospital readmission. The baseline characteristics of the readmitted and non-admitted cohorts are detailed in Table 1.

### 3.2. Preoperative Characteristics

The mean age of readmitted patients was slightly higher than that of non-readmitted patients (31.44 ± 5.94 years vs. 30.74 ± 5.62 years, *p* < 0.001). BMI was also significantly higher in the readmitted group (36.11 ± 8.43 kg/m^2^ vs. 34.31 ± 7.26 kg/m^2^, *p* < 0.001). A significantly higher proportion of Black patients were noted in the readmission group (17.99% vs. 9.83%, *p* < 0.001). The prevalence of comorbid conditions such as diabetes (11.03% vs. 8.19%, *p* < 0.001) and hypertension (11.49% vs. 4.68%, *p* < 0.001) was notably higher among readmitted patients as well.

Postoperative outcomes are presented in Table 2. The mean operative time was significantly longer for patients who were readmitted (57.42 ± 35.84 min vs. 51.71 ± 28.95 min, *p* < 0.001). Several postoperative complications were significantly more frequent in the readmitted cohort, including organ/surgical site infections (12.17% vs. 0.5%, *p* < 0.001), wound infections (3.75% vs. 0.09%, *p* < 0.001), and pneumonia (2.22% vs. 0.11%, *p* < 0.001). The readmitted group also had higher rates of thromboembolic events such as pulmonary embolism (1.61% vs. 0.06%, *p* < 0.001) and deep vein thrombosis (1.07% vs. 0.08%, *p* < 0.001). The initial total hospital length of stay was also longer for the readmitted group (3.6 ± 3.86 days vs. 2.98 ± 2.6 days, *p* < 0.001).

### 3.3. Logistic Regression

**Figure 2:**
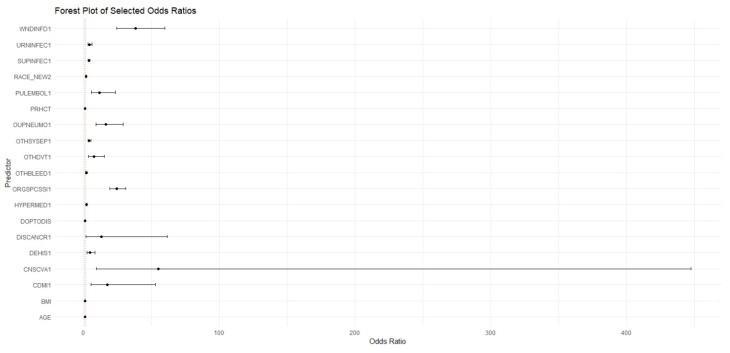
Forest Plot of Odds Ratios of Statistically Significant Predictors Computed from Logistic Regression Model. (WNDINFD1: Wound Disruption; URNINFEC1: Urinary Tract Infection; SUPINFEC1: Superficial Surgical Site Infection; RACE_NEW2: African American Race; PULEMBOL1: Pulmonary Embolism; PRHCT: Preoperative Hematocrit; OUPNEUMO1: Pneumonia; OTHSYSEP1: Sepsis; OTHDVT1: Deep Vein Thrombosis Requiring Therapy; OTHBLEED1: Bleeding Requiring a Transfusion (within 72 h); ORGSPCSSI1: Organ/Space Surgical Site Infection; HYPERMED1: Hypertension Requiring Medication; DOPTODIS: Days from Operation to Discharge; DISCANCR1: Disseminated Cancer; DEHIS1: Wound Disruption; CNSCVA1: Cerebrovascular Accident (Stroke); CDMI1: Myocardial Infarction; BMI: Body Mass Index; AGE: Age).

The Forest plot of ORs (Figure 2) identifies significant predictors using the LR model. Preoperative predictor variables of readmission included age (OR: 1.03; 1.02–1.04, *p* < 0.001), BMI (OR: 1.01; 1.00–1.02, *p* = 0.0036), Black race (OR: 1.85; 1.53–2.25, *p* < 0.001), hypertension (OR: 1.94; 1.57–2.37, *p* < 0.001), and cancer (OR: 12.90; 1.72–61.5, *p* = 0.003).

### 3.4. Extreme-Gradient Boosting

**Figure 3:**
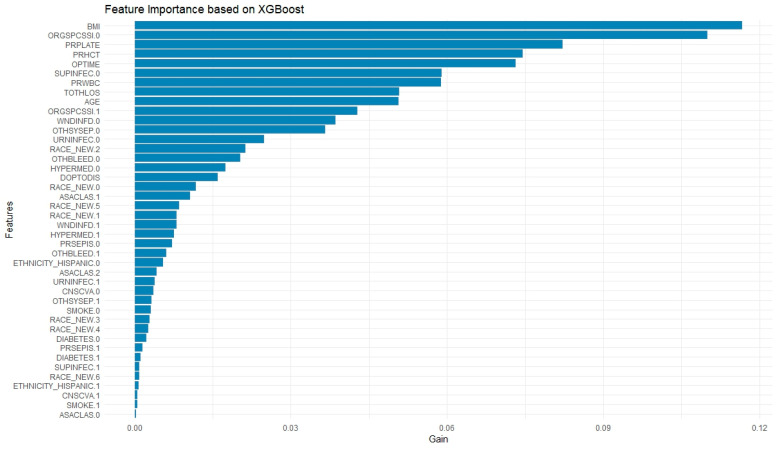
Feature Importance Plot from XGBoost Model. (BMI: Body Mass Index; ORGSPCSSI: Organ/Space Surgical Site Infection; PRPLATE: Preoperative Platelet Count; PRHCT: Preoperative Hematocrit; OPTIME: Total Operation Time (minutes); SUPINFEC: Superficial Surgical Site Infection; PRWBC: Preoperative White Blood Cell Count; TOTHLOS: Total Hospital Length of Stay; AGE: Age (in years, top-coded at 90+); WNDINFD: Deep Incisional Surgical Site Infection; OTHSYSEP: Sepsis; URNINFEC: Urinary Tract Infection; RACE_NEW: Race (categorical variable); OTHBLEED: Bleeding Requiring a Transfusion (within 72 h); HYPERMED: Hypertension Requiring Medication; DOPTODIS: Days from Operation to Discharge; ASACLAS: ASA Physical Status Classification; PRSEPIS: Preoperative Sepsis; CNSCVA: Cerebrovascular Accident (Stroke); SMOKE: Smoking Status (within 1 year); ETHNICITY_HISPANIC: Hispanic/Latino Ethnicity; DIABETES: Diabetes Mellitus).

Preoperative predictors identified by XGBoost included BMI (Feature Importance: 0.117), preoperative platelet count (0.082), preoperative hematocrit (0.075), preoperative white blood cell count (0.059), age (0.051), Black race (0.021), and a history of bleeding disorders (0.020). Perioperative predictors of readmission included operation time (0.073), total hospital length of stay (0.051), organ/surgical site infection (0.043), wound infection (0.039), sepsis (0.037), and urinary tract infection (0.025) (Figure 3).

### 3.5. Random Forest

**Figure 4:**
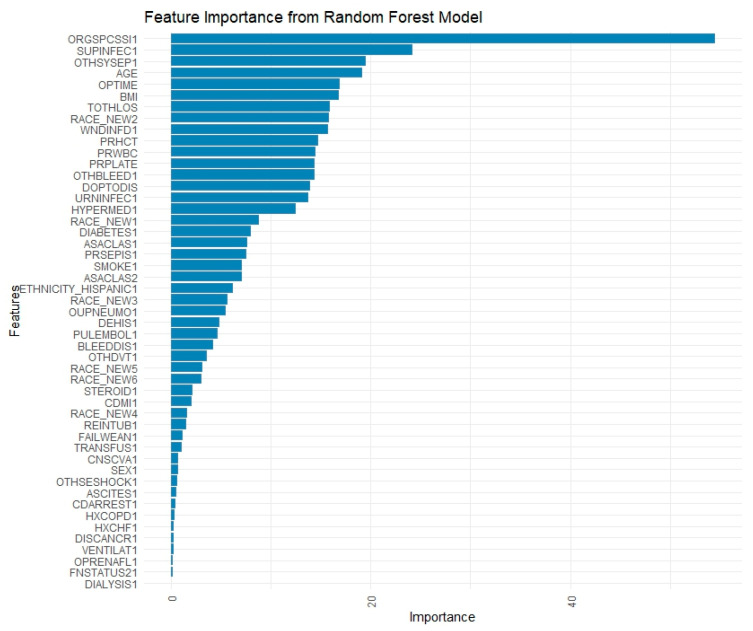
Feature Importance Plot from Random Forest Model Using All Predictor Variables. (BMI: Body Mass Index; ORGSPCSSI: Organ/Space Surgical Site Infection; PRPLATE: Preoperative Platelet Count; PRHCT: Preoperative Hematocrit; OPTIME: Total Operation Time (minutes); SUPINFEC: Superficial Surgical Site Infection; PRWBC: Preoperative White Blood Cell Count; TOTHLOS: Total Hospital Length of Stay; AGE: Age (in years, top-coded at 90+); WNDINFD: Deep Incisional Surgical Site Infection; OTHSYSEP: Sepsis; URNINFEC: Urinary Tract Infection; RACE_NEW: Race (categorical variable); OTHBLEED: Bleeding Requiring a Transfusion (within 72 h); HYPERMED: Hypertension Requiring Medication; DOPTODIS: Days from Operation to Discharge; ASACLAS: ASA Physical Status Classification; PRSEPIS: Preoperative Sepsis; CNSCVA: Cerebrovascular Accident (Stroke); SMOKE: Smoking Status (within 1 year); ETHNICITY_HISPANIC: Hispanic/Latino Ethnicity; DIABETES: Diabetes Mellitus).

**Figure 5:**
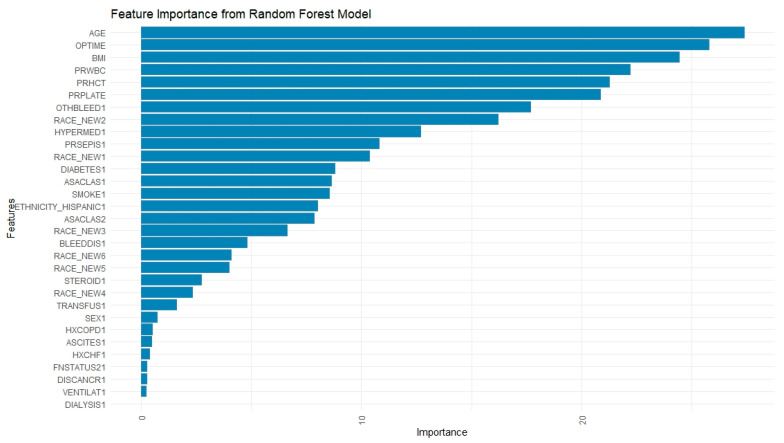
Feature Importance Plot from Random Forest Model Using Only Pre-operative Variables. (BMI: Body Mass Index; ORGSPCSSI: Organ/Space Surgical Site Infection; PRPLATE: Preoperative Platelet Count; PRHCT: Preoperative Hematocrit; OPTIME: Total Operation Time (minutes); SUPINFEC: Superficial Surgical Site Infection; PRWBC: Preoperative White Blood Cell Count; TOTHLOS: Total Hospital Length of Stay; AGE: Age (in years, top-coded at 90+); WNDINFD: Deep Incisional Surgical Site Infection; OTHSYSEP: Sepsis; URNINFEC: Urinary Tract Infection; RACE_NEW: Race (categorical variable); OTHBLEED: Bleeding Requiring a Transfusion (within 72 h); HYPERMED: Hypertension Requiring Medication; DOPTODIS: Days from Operation to Discharge; ASACLAS: ASA Physical Status Classification; PRSEPIS: Preoperative Sepsis; CNSCVA: Cerebrovascular Accident (Stroke); SMOKE: Smoking Status (within 1 year); ETHNICITY_HISPANIC: Hispanic/Latino Ethnicity; DIABETES: Diabetes Mellitus).

Per the RF feature importance plot (Figure 4), organ/surgical site infections (importance score: 54.67), surgical site infection (24.62), operation to discharge time (21.49), and sepsis (20.46) were amongst the strongest postoperative predictive factors of readmission. When using only pre- and intra-operative variables to train and test the model, the most powerful predictors included age (27.39), operation time (25.81), BMI (24.44), preoperative white blood cell count (22.25), hematocrit (21.30), and platelet count (20.87). Other significant factors were a history of bleeding disorders (17.71), Black race (16.22), and hypertension (12.72) (Figure 5).

### 3.6. Model Performance

**Figure 6:**
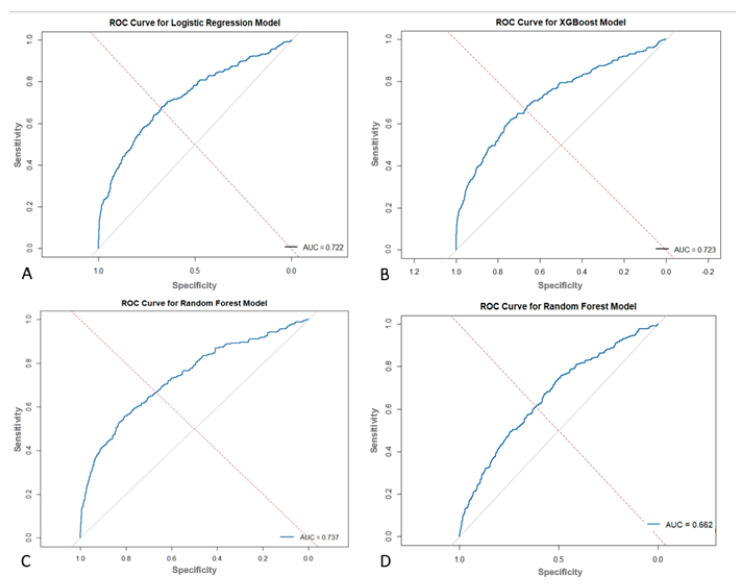
Receiver Operating Characteristic (ROC) Curves for (**A**) Logistic Regression, (**B**) Extreme Gradient Boosting (XGBoost) and (**C**) Random Forest. (**D**) Random Forest ROC Curve Using Only Pre-Operative Variables.

The RF model had the best performance in predicting readmission following C-sections with an AUC of 0.737 compared to XGBoost (AUC = 0.723) and LR (AUC = 0.722) (Figure 6). The confusion matrices for training and testing data are presented in Table 3, with confidence intervals denoted in brackets. The RF model demonstrated the highest sensitivity (72.03%) and specificity (61.33%) in the testing set. The LR model had a sensitivity of 49.4% and specificity of 82.4% in the testing set, and the XGBoost model had a sensitivity of 75.9% and specificity of 55.6% in the testing set. The RF model limited to perioperative data only demonstrated an AUC of 0.662 with a sensitivity of 83.14% and specificity of 34.47% in the testing set. On the test set, per McNemar’s test, LR made significantly fewer discordant errors than RF (χ^2^ ≈ 1017, *p* < 0.001) and XGBoost (χ^2^ ≈ 1529, *p* < 0.001). RF outperformed XGBoost (χ^2^ ≈ 69, *p* < 0.001), and the pre-operative-only RF made significantly more errors than all other models (all *p* < 0.001).

## 4. Discussion

To our knowledge, the current study is the first to describe the use of ML to predict 30-day readmission rates for individuals undergoing C-section in the US using a large national database. We demonstrated that ML algorithms trained on readily available preoperative clinical data could predict the rate of readmission following C-section, with a sensitivity up to 83.14%. To date, many studies have noted C-section characteristics and complications that are associated with readmissions following C-section delivery; however, ML tools, which provide more powerful and dynamic predictive capabilities, have not been widely used in this area.

A similar study by Firouzbakht et al. was recently published, where different models were built to predict postpartum readmission rates in Iranian women [4]. Per their multivariate analysis, they found that the method of labor pain onset (e.g., labor pain timing based on the mode of delivery), mode of delivery, and intrapartum complications were the strongest predictors of readmission following childbirth [4]. An ML model was also built using RF, which found labor pain onset, gravidity, and birth weight to be the most predictive of readmission based on their importance features plot. However, the study does not report the performance of their models using AUC or confusion matrices (e.g., sensitivity, specificity, etc.) and does not compare the performance of the ML to other basic non-ML statistics models they built, which limits the interpretability of their models.

The present study found that ML models outperform standard statistical measures in predicting 30-day readmission using both a combination of pre, intra, and postoperative variables as well as only preoperative variables. With all variables combined, the RF model outperformed the XGBoost and LR models in overall predictive accuracy (AUC = 0.737 vs. 0.723 vs. 0.722) with a sensitivity of 72.03%. Operation time as well as postoperative complications, mainly surgical site infections and sepsis, were strong predictors of readmission in these ML models. A separate RF model with only preoperative variables was built which achieved moderate predictive accuracy of 0.662 and a sensitivity of 83.14%, which highlights its ability to predict true readmissions in the cohort. The purpose of building this model with only preoperative variables is to show how ML can be easily used in the preoperative setting to risk stratify patients prior to their C-section using data that is readily available about them. This would not only contribute to a more comprehensive pre-surgical evaluation, but would also allow for proactive postsurgical planning and appropriate management for patients at high risk of readmission.

Our findings align with several studies that have shown that pregnancy complications and maternal comorbidities increase the rate of readmission following childbirth [13,14,15]. One study identified the most common early postpartum readmission risk factors (defined as within 6 days postpartum) as sepsis, severe maternal morbidity, and preeclampsia before birth [16]. There was also a notable 2-fold higher risk for readmission among patients with preterm birth <34 weeks gestation or a major mental health condition [16]. Postpartum readmission following C-section not only presents significant clinical challenges but also imposes significant economic burdens on the healthcare system. Readmissions often include extended hospital stays, increased utilization of resources, and overall increased healthcare costs for both the individual patient and the hospital. For example, among 11.8 million US Medicare beneficiaries who were discharged from a hospital in 2003 to 2004, >2.3 million (19.6%) were rehospitalized within 30 days with an estimated cost of over $17 billion [6]. It is clear that patients, healthcare systems, and providers should all aim to avoid unnecessary readmissions while simultaneously ensuring patient safety. Identification of patients at risk of immediate return to the hospital after birth could help in identifying those needing additional preparedness for discharge and thus minimizing the need for readmission [16].

The clinical relevance of ML models lies in their ability to perform complex modeling to identify patients at risk of readmission and help to tailor discharge and follow-up care, thereby increasing overall patient satisfaction and patient outcomes as well as reducing healthcare costs. With a low event rate (2.39%), models with higher specificity (e.g., LR) yield fewer overall classification errors at the pre-specified operating points, whereas RF/XGBoost achieve higher sensitivity at the cost of more false positives. This trade-off is also reflected in PPV/NPV and can be tuned via alternative thresholds. We acknowledge that the low specificity (34.47%) of the pre-operative model is a significant trade-off for its high sensitivity, which translates to a high false-positive rate. Therefore, its intended clinical application is not as a standalone diagnostic predictor, but as a screening tool to flag patients for low-intensity interventions. For example, a high-risk flag could trigger enhanced discharge education or an automated follow-up call, where the cost of a false positive is minimal compared to the clinical and financial burden of an unforeseen readmission. ML models have been utilized in various other specialties, specifically neurosurgery and thoracic surgery, to predict post-surgical readmission rates, all with an AUC > 0.70 [7,17]. ML can be incorporated to increase pre-surgical evaluation of patients to identify risk factors and predictors of readmission to the hospital following delivery, thus assisting in shared decision-making, resource allocation, and quality improvement.

### Strengths and Limitations

The study utilizes a large, nationwide database that provides access to a large and diverse population of patients across institutions within the US, thus improving the robustness of the study’s findings and allowing a representative sample of patients undergoing C-section. The use of real-world clinical data enhances the study’s relevance to clinical practice as well. Utilization of ML algorithms to predict 30-day readmission is a novel approach compared to more traditional statistical methods, and this study demonstrates the potential of ML in healthcare to improve outcomes. We utilize internal validation through the testing sets to ensure the reliability of the models and provide consistent predictions within the dataset.

However, this study has several limitations that should be considered when interpreting the findings. The retrospective nature of the study limits the ability to establish causality between observed associations. Hospitals that are not participating in the NSQIP database have been excluded, and thus, input from smaller hospitals or those in rural areas may have different resources, staffing and care patterns that could affect readmission rates but are not reflected in the NSQIP database. The multi-center design could pose a limitation as well, as NSQIP spans over 700 hospitals but is split by patient rather than by hospital site, which limited our ability to carry out a hospital-stratified validation. There is also limited use of socioeconomic and behavioral health data in predicting readmissions as this data was not available in the database, which may play a role in many populations. Similarly, factors such as social determinants of health, patient support networks and outpatient follow up are often not included in the NSQIP clinical and perioperative data. These factors can heavily influence readmission risk, limiting the model’s application to certain real-world settings. Regarding the ML model that was created using only preoperative data, the RF showed great potential in its ability to flag patients as potential readmissions due to its relatively high sensitivity (83.14%), yet its low specificity (34.47%) denotes that it may have challenges in recognizing patients who will not be readmitted. This could potentially contribute to alert fatigue among clinicians and must be carefully considered before any real-world implementation. However, many published risk scores, including the Wells Score for pulmonary embolism, have a high sensitivity and low specificity yet are reliably used for detection of certain conditions [18]. The notable gap between training (AUC 0.957) and testing (AUC 0.737) performance in the RF model suggests a degree of overfitting, where the model learned patterns specific to the training data that did not fully generalize. While 10-fold cross-validation and hyperparameter tuning was done to mitigate this, the complexity of the model likely contributed to this discrepancy. Therefore, the testing set results should be considered the primary indicator of real-world performance. Overfitting was addressed through 10-fold cross-validation during model training, grid search-based hyperparameter tuning, and evaluation on an independent test set. Additionally, early stopping (for XGBoost) and threshold optimization techniques were incorporated to further promote model robustness. Another limitation of this study is the inherent class imbalance in the outcome variable, with only 2.39% of patients experiencing 30-day readmission. While we accounted for this in the XGBoost model using the scale_pos_weight parameter to rebalance the class distribution during training, we did not apply additional balancing techniques such as oversampling, undersampling, or synthetic data generation. This was done to preserve the nature of the data and provide a representative predictive tool based on real clinical values.

Our internal validation relies on a single stratified 80/20 train-test split. While standard practice, this approach can be subject to partitioning variance, and model performance could differ slightly with a different random split. While this provides a consistent estimate of internal model performance, the absence of external or temporal validation limits the generalizability of our findings. The notable gap between training and testing AUC in the Random Forest model (0.957 vs. 0.737) also suggests potential model optimism despite cross-validation and tuning. Future work will include external datasets or time-split validation to better assess real-world performance.

Future research should be aimed at improving predictive ML models through integration of additional variables such as patient socioeconomic status, mental health factors, or geographic variations with validation on external cohorts. In addition, the hope is to use the ML models to develop a risk calculator that can be used at the bedside or integrated into EHRs where C-section patients are assigned percentage risk of readmission automatically.

## 5. Conclusions

Machine learning can be used to identify patients at a high risk of readmission after C-section using only preoperative data. Ability to identify patients at risk can prompt possible interventions during initial admission or during follow up to prevent readmission, thus alleviating economic burdens on the healthcare system and ultimately improving patient outcomes.

## Figures and Tables

**Table 1:** Pre-Operative Baseline Characteristics of Patients Undergoing C-Section, 2012–2022 (n = 54,593).

Parameter	Non-Readmitted (n = 53,287)	Readmitted (n = 1306)	*p*-Value
Mean Age (years)	30.74 ± 5.62	31.44 ± 5.94	<0.001
Mean Body Mass Index (kg/m^2^)	34.31 ± 7.26	36.11 ± 8.43	<0.001
Race, White	50.21%	47.01%	<0.001
Race, Black or African American	9.83%	17.99%	<0.001
Race, Asian	6.26%	6.13%	0.335
Native Hawaiian or Other Pacific Islander	0.56%	0.54%	
Race, American Indian or Alaska Native	1.41%	1.15%	0.234
Race, Other	1.91%	1.68%	
Hispanic Ethnicity	13.29%	12.33%	0.331
Diabetes	8.19%	11.03%	<0.001
Smoking	8.67%	10.87%	0.006
Ventilator Usage	0.05%	0.15%	0.260
Chronic Obstructive Pulmonary Disease	0.03%	0%	1.000
Congestive Heart Failure	0.06%	0.46%	<0.001
Hypertension	4.68%	11.49%	<0.001
Dialysis	0.02%	0%	1.000
Cancer	0.01%	0.15%	0.002
Steroid Use	0.47%	0.77%	0.182
Bleeding Disorders	1.28%	1.91%	0.061
Transfusions	0.23%	0.77%	<0.001
Sepsis	8.04%	12.79%	<0.001
ASA Class 1–2	78.87%	68.53%	<0.001
ASA Class > 2	20.53%	30.78%	<0.001
Mean Hematocrit (%)	35.75 ± 3.45	35.35 ± 3.68	0.00
Mean White Blood Cell Count (10^3^/µL)	10.51 ± 3.25	10.63 ± 3.52	0.199
Mean Platelet Count (10^3^/µL)	226.2 ± 63.01	224.44 ± 65.75	0.338

**Table 2:** Post-Operative Characteristics of Patients Undergoing C-Section, 2012–2022 (n = 54,593).

Parameter	Non-Readmitted (n = 53,287)	Readmitted (n = 1306)	*p*-Value
Mean Operation Time (Minutes)	51.71 ± 28.95	57.42 ± 35.84	<0.001
Organ/Surgical Site Infection	0.5%	12.17%	<0.001
Wound Infection	0.09%	3.75%	<0.001
Dehiscence	0.17%	2.37%	<0.001
Pneumonia	0.11%	2.22%	<0.001
Reintubation	0.07%	1%	<0.001
Pulmonary Embolism	0.06%	1.61%	<0.001
Failure to Wean	0.06%	0.84%	<0.001
Urinary Tract Infection	0.84%	4.59%	<0.001
Bleeding Requiring a Transfusion (within 72 h)	3.19%	9.65%	<0.001
Deep Vein Thrombosis	0.08%	1.07%	<0.001
Sepsis	1.14%	8.65%	<0.001
Requiring Return to Operating Room	0.59%	13.94%	<0.001
Total Hospital Length of Stay	2.98 ± 2.6	3.6 ± 3.86	<0.001

**Table 3:** Confusion Metric Reports on Training and Testing Data for 30-Day Readmission in Patients who Underwent C-Section (2012–2022).

Metric	Outcome	Logistic Regression (LR)	XGBoost	Random Forest (RF)	RF with only Perioperative Data
AUC	Training	0.748 [0.731–0.765]	0.773 [0.756–0.790]	0.957 [0.948–0.966]	0.866 [0.852–0.880]
	Testing	0.722 [0.706–0.738]	0.723 [0.690–0.756]	0.737 [0.703–0.771]	0.662 [0.626–0.699]
Sensitivity	Training	0.0957 [0.078–0.114]	0.7943 [0.770–0.819]	0.8727 [0.852–0.893]	0.9254 [0.909–0.941]
	Testing	0.4943 [0.434–0.555]	0.7586 [0.707–0.811]	0.7203 [0.666–0.775]	0.8314 [0.786–0.877]
Specificity	Training	0.9985 [0.998–0.999]	0.5844 [0.580–0.589]	0.8459 [0.843–0.849]	0.5474 [0.543–0.552]
	Testing	0.8236 [0.816–0.831]	0.5556 [0.546–0.565]	0.6133 [0.604–0.623]	0.3447 [0.336–0.354]

## Data Availability

The data used in this study were obtained from the American College of Surgeons National Surgical Quality Improvement Program (NSQIP).
